# qPCR-based relative quantification of the brown algal endophyte *Laminarionema elsbetiae* in *Saccharina latissima*: variation and dynamics of host—endophyte interactions

**DOI:** 10.1007/s10811-017-1367-0

**Published:** 2017-12-19

**Authors:** Miriam Bernard, Sylvie Rousvoal, Bertrand Jacquemin, Marion Ballenghien, Akira F. Peters, Catherine Leblanc

**Affiliations:** 1 CNRS, UMR 8227, Integrative Biology of Marine Models, Station Biologique de Roscoff, Sorbonne Universités, UPMC Univ. Paris 06, 29680 Roscoff, France; 2 CNRS, UMI 3614, Evolutionary Biology and Ecology of Algae, Station Biologique de Roscoff, Sorbonne Universités, UPMC Univ. Paris 06, 29680 Roscoff, France; 3 Present Address: CEVA, 22610 Pleubian, France; 4 Bezhin Rosko, 29250 Santec, France

**Keywords:** Endophytes, *Laminarionema elsbetiae*, Kelps, *Saccharina latissima*, Quantitative PCR

## Abstract

Morphological changes—such as dark spots, twisted stipes and deformed blades—have been observed in wild and cultivated *Saccharina latissima*. The putative cause for the disease symptoms is the filamentous endophytic brown alga *Laminarionema elsbetiae*, which is known to invade stipes and fronds of its hosts. Little is known about this interaction and its occurrence in the field, although former studies indicated high endophyte prevalence in kelp populations. Previous epidemiological studies on kelp endophytes were mainly based on the examination of microscopic sections, followed by time-consuming isolation and cultivation steps in order to identify the endophyte and a reliable method to quantify endophyte infections was missing. As a novel approach, we established and validated a qPCR assay for relative quantification of the endophyte *L. elsbetiae* within its host *S. latissima*, which allows to examine both, the prevalence of endophytic algae and the severity of infections. The assay was shown to be highly specific and suitable to reliably detect small amounts of endophyte DNA in the host. Using this method, we detected very high endophyte prevalence in the investigated kelp populations, up to 100% in young *S. latissima* sporophytes in Brittany during spring. Furthermore, our results suggest that *Saccharina* sporophytes are infected early in their life and that seasonality and environmental factors have a significant impact on infection rates. In the future, this approach could also be applied to study other host-endophyte pairs using specific primers.

**Electronic supplementary material:**

The online version of this article (10.1007/s10811-017-1367-0) contains supplementary material, which is available to authorized users.

## Introduction

The sugar kelp *Saccharina latissima* (Laminariales, Phaeophyceae) is an important primary producer in temperate to cold northern hemisphere coastal ecosystems (Bartsch et al. 2008) and an economically relevant seaweed with high industrial potential (Østgaard et al. 1993; Adams et al. 2009). Growing or being cultivated in the sea *S. latissima* is exposed to a high number of potentially harmful organisms such as fungi, bacteria or endophytic algae (Andrews 1977; Wu et al. 1983; Apt 1988a; Potin et al. 2002). Previous studies on the latter reported a high prevalence of filamentous endophytic algae in kelp populations, with up to 100% of infected individuals (Andrews 1977; Lein et al. 1991; Peters and Schaffelke 1996; Schaffelke et al. 1996; Ellertsdóttir and Peters 1997). Amongst them is *Laminarionema elsbetiae* (Ectocarpales, Phaeophyceae), a filamentous brown algal endophyte highly prevalent in European wild *S. latissima* populations (Peters and Ellertsdóttir 1996; Ellertsdóttir and Peters 1997). It invades stipes and fronds of its host, thereby potentially not only causing morphological changes but even more severe impairment as it has been shown for other filamentous endophytic brown algae (Yoshida and Akiyama 1979; Apt 1988a, b; Peters and Schaffelke 1996; Ellertsdóttir and Peters 1997; Thomas et al. 2009). Despite an increasing interest in this topic due to the economic importance of *Saccharina* aquaculture (Chen 2004), little is known about this particular interaction, its prevalence in the field, the natural infection process and variation under different environmental conditions. A considerable drawback is the lack of a common comparable and rapid method to conduct these studies. In particular, there is no reliable technique to quantify endophyte infections, which is crucial to investigate the dynamics of this phenomenon.

Quantitative PCR is a well-established tool for the detection of pathogens in the field of plant-pathogen interactions (Brouwer et al. 2003; Gachon et al. 2004) which has lately also been applied for the detection of the pathogenic oomycete *Eurychasma dicksonii* in *Ectocarpus siliculosus* (Gachon et al. 2009). Here, we developed a highly specific qPCR assay that is not only fast and reproducible but also suitable to detect minor amounts of target DNA. This method allows us to examine the prevalence of endophyte infections, i.e. the number of infected thalli in a population, and the severity of infection, i.e. the relative amount of endophyte present in the host tissue. The first aim of this study was to validate this qPCR assay according to recommended guidelines (Bustin et al. 2009). Subsequently, we applied the assay to examine the distribution of filaments of *L. elsbetiae* along the thallus of *S. latissima* and the impact of seasonality and geographic variation on endophyte infection rates in different kelp populations. The natural infection process was studied by cultivating laboratory-grown *S. latissima* sporophytes in a seaweed farm and comparing their infection rates with those of wild individuals. Finally, the assay was applied to assess the specificity of *L. elsbetiae* towards different kelp species.

## Material and methods

### In situ algal sampling

To determine the distribution of endophyte occurrence along the thallus, tissue was punched out (ø 2.8 cm) at four positions on *S. latissima* sporophytes: (1) 50% of the stipe length (piece of 2.8 cm length), (2) 10% of the blade length, (3) 50% of the blade length, and (4) 90% of the blade length. Samples of *S. latissima* were collected in the same location of different populations, i.e. in Northern Brittany (Perharidy near Roscoff; 48.73° N, 4.00° W, *N* = 10) in March and April 2016, in Southern Brittany (Locmariaquer; 47.55° N, 2.92° W, *N* = 5) in March 2016, and in Western Scotland (Bridge over the Atlantic; 56.31° N, 5.58° W, *N* = 5) in April 2016. Additionally, two sections were made next to each punch-out to look for the presence of endophytic filaments using a light microscope.

For the following studies, all samples were taken from the distal part of the blades (i.e. 90% of the blade length) of the kelp sporophytes. The onset of endophyte infections in the field was explored by obtaining infection rates of young *S. latissima* sporophytes with different thallus lengths collected in March 2017 in Northern Brittany (Perharidy; 48.73° N, 4.00° W, *N* = 10) that were grouped according to the host length: 3–5 cm (*N* = 6), 6–10 cm (*N* = 7), 11–15 cm (*N* = 8), 16–26 cm (*N* = 4), and > 26 cm (*N* = 30).

An experimental set-up was used to investigate the impact of natural infection on laboratory-grown sporophytes. Gametophytes descending from spores of *S. latissima* from Perharidy were seeded on collectors in February 2016 by submerging them in 50-mL Falcon tubes overnight. Then, the collectors were transferred to filtered seawater containing half-strength Provasoli enrichment (10 mL solution per L seawater, Provasoli 1968). The young sporophytes were grown in 11 °C with 40 μmol photons m^−2^ s^−1^ with a light/dark cycle of 8/16 h during the first 20 days and 12/12 h until the end of the experiment. After 68 days, when the sporophytes had reached a length of 2–4 cm, a part of them was transferred to a seaweed farm in vicinity to the wild population (4 km distance) in April 2016 while the rest was maintained in laboratory conditions. In October 2016, infection rates in samples from the individuals cultivated in the farm (*N* = 57) were compared to samples collected from the wild population at Perharidy (*N* = 30) within the same week. The kelps kept in laboratory conditions since April 2016 served as controls (*N* = 27).

Seasonal variation of endophyte infection rates was examined in *S. latissima* sporophytes collected in March 2016, April 2016, July 2016, September 2016, October 2016, November 2016, December 2016, February 2017 and March 2017 (*N* = 30, each month) in Perharidy.

*Saccharina latissima* samples were collected in Southern Brittany in March 2016 (*N* = 12) and in Western Scotland in April 2016 (*N* = 30) and compared to the samples collected in Perharidy in March and April 2016 (*N* = 30, each) to investigate geographic variation of endophyte infection rates.

To explore host specificity of *L. elsbetiae*, tissue was punched out from distal parts of blades in *L. digitata*, *L. hyperborea* and *L. ochroleuca* (*N* = 10 for each species) collected in March 2017 in Perharidy and compared to the infection rate of *S. latissima* (*N* = 30). Additionally, 10 L seawater (*N* = 3) was collected and directly filtered through a 150-μm mesh. Subsequently, the water was filtered through 3-μm polycarbonate filters (Nucleopore Track-Etched Membranes, Whatman, GE Healthcare, USA) with a vacuum pump. The filters were transferred to cryotubes, frozen in liquid nitrogen and kept in − 20 °C until DNA was extracted.

All samples were collected haphazardly regardless of possible morphological infection symptoms. The punched-out tissue was soaked dry with tissue paper, transferred to silica gel and stored in silica until DNA extraction.

### Monospecific algal cultures

DNA from monospecific algal cultures was used for setting-up and validating the qPCR assay. The cultures of laboratory-grown kelps were started from freshly released spores of mature sporophytes collected at Perharidy. Developing sporophytes were kept in 10-L bottles containing half-strength Provasoli enrichment (10 mL Provasoli solution/L seawater) in 14 °C and ~ 20 μmol photons m^−2^ s^−1^ at 12-h light/day with weekly changes of the culture medium. Cultures of the filamentous brown algal endophyte *L. elsbetiae* were grown from the strain LelsPH14-01 obtained from the Bezhin Rosko culture collection (origin Perharidy, France). Isolation of other algal strains from the order Ectocarpales was performed as described by Peters (1991). Ectocarpales cultures were kept in 14 °C and 5 μmol photons m^−2^ s^−1^ at 12-h light/day with monthly changes of the culture medium (half-strength Provasoli enrichment). The cultured algal material was soaked dry with tissue paper and freeze-dried for DNA extraction.

### DNA extraction

All samples were extracted according to the same protocol to limit differences in extraction yields. The dried algal material was ground in a mechanical bead grinder (Tissuelyser II, Qiagen, Germany) twice for 2 min at 30 Hz. Twenty milligrams of ground material was transferred to a 2-mL Eppendorf tube and used for the following DNA extraction that was adapted after Apt et al. (1995). One millilitre of extraction buffer (100 mM Tris-HCl [pH 7.5], 1.5 M NaCl, 2% CTAB, 50 mM EDTA [pH 8], 50 mM DTT) was added to the ground tissue, and samples were incubated at room temperature on a shaker at 250 rpm for 1 h. One vol of chloroform/isoamyl alcohol (24:1) was added, and the two phases were mixed by vortexing and centrifuged at 10.000 rpm for 15 min. The upper phase was transferred to a new tube and 0.3 vol ethanol was added drop by drop until polysaccharide precipitation was visible, followed by a second chloroform extraction and another centrifugation step at 10.000 rpm for 15 min. The upper phase was transferred onto the filter of the Nucleospin plant II kit (Macherey-Nagel, Germany), and the protocol recommended by the manufacturer was followed from this step onwards with two elution steps of 50 μL.

For DNA extraction from the seawater samples, 1.5 mL of lysis buffer (0.7 M sucrose, 50 mM Tris pH 8, 40 mM EDTA) was added to each filter. One hundred microliter lysozyme (20 mg mL^−1^) was added, and samples were shaken at 37 °C for 45 min. Twenty microliter proteinase K (20 mg mL^−1^) and 100 μL 20% SDS was added, and samples were homogenised by inversion and incubated at 55 °C for 1 h. The content was transferred to a new tube and 1 vol phenol-chloroform-isoamyl alcohol (25:24:1) was added; the phases were mixed and then centrifuged for 15 min at 4500 rpm at 4 °C. The supernatant was mixed with 1 vol of the binding buffer from the Nucleospin plant II kit (Macherey-Nagel, Germany), and the protocol recommended by the manufacturer was followed from this step onwards with two elution steps of 50 μL.

DNA concentrations were measured with a Qubit Fluorometer (Thermo Fisher, USA) and diluted to 0.05 ng DNA μL^−1^ with autoclaved milliQ-filtered H_2_O.

### qPCR and evaluation of the assay

The first primer pair CG64 and CG65 (Gachon et al. 2009) matched the 18S rDNA of all Ectocarpales and Laminariales (72-bp amplicon size) and was used to amplify 18S rDNA from both, host and endophyte DNA. The second primer pair LelsITS1-F2 (TTTCGAGAGCTTTCGAGAGG) and LelsITS1-R2 (TCTTCACGCCTCTTACATGG) (83-bp amplicon size) was designed to specifically match the partial ITS1 of *Laminarionema elsbetiae*. Specificity of the latter primer pair was tested by blasting the sequence and testing it with the DNA from 10 other brown algae diluted to 0.05 ng DNA μL^−1^, including algal endophytes from the order Ectocarpales and possible hosts from the order Laminariales (Fig. S1). The qPCR products were run in a 2.5% agarose gel electrophoresis at 100 V for 25 min to check for presence or absence of bands.

Artificial mixtures of host and endophyte DNA were assembled to assess if different amounts of endophyte DNA were detectable reliably. Therefore, 1 ng of DNA from *S. latissima* was mixed with 0.0024, 0.012, 0.06 and 0.3 ng of DNA from *L. elsbetiae*.

Standard curves for the CG primer pair were constructed in triplicates with 1:5 serial dilutions of *S. latissima* DNA, extracted from a laboratory-grown sporophyte, ranging from a concentration of 0.5 ng to 6.4 × 10^−6^ ng. Standard curves for the LelsITS1 primer pair were constructed with 1:2 serial dilutions of *L. elsbetiae* DNA, extracted from the strain LelsPH14-01, ranging from a concentration of 0.375 ng to 1.14 × 10^−5^ ng.

A total of 2.5 μL LightCycler 480 SYBR Green I Master (2x, Roche Diagnostics, Germany) was mixed with the primers (400 nM), and 2.9 μL of this mix was added to 2.1 μL of diluted DNA (0.05 ng μL^−1^). Real-time PCR was performed on a LightCycler 480 (Roche Life Science, Germany) in white 384-well plates, sealed with adhesive foil. A 5-min denaturation step at 95 °C was followed by 55 cycles of 10 s at 95 °C and 15 s at 60 °C and 15 s at 72 °C. After each run, a dissociation curve was obtained by heating the samples from 65 to 97 °C. The dissociation curves indicated a single product for both primer pairs (data not shown). All samples were run in triplicates, as recommended by Pfaffl (2004), and autoclaved milliQ H_2_O was used as negative control. For relative quantification, the differences between the quantification cycles (ΔC_q_) obtained by two qPCRs with the different primer pairs run in parallel on the same DNA sample were measured, as by Gachon et al. (2009). The resulting ΔC_q_ values correlate negatively to the relative amount of endophyte DNA in the sample.

No relative quantification was performed for the water samples. Only the *L. elsbetiae*-specific primer pair was used in a qPCR reaction, and the final qPCR product was run in a 2.5% agarose gel electrophoresis to check for presence or absence of endophyte DNA.

### Data analysis

Cycle thresholds were calculated with the LightCycler 480 Software (Roche, Germany) and exported to Excel 2013 (Microsoft, USA) where ΔC_q_ values of each DNA sample were determined. Values are reported as average ± standard deviation. Graphs of the standard curves were drawn with GraphPad Prism (GraphPad Prism Software, Inc., USA), and the heat map was constructed in R Studio (RStudio, Inc., USA). SPSS (IBM Corp. Released 2013. IBM SPSS Statistics for Windows, Version 22.0. Armonk, NY: IBM Corp.) was used to perform statistical analyses. Normality of the data was tested with the Shapiro-Wilk test and homogeneity of variances with the Levene test. Data with normal distribution and homogeneous variances was analysed with one-way ANOVA. In the case of heterogeneous variances, the non-parametric Kruskal-Wallis test was used.

## Results

### Set-up and validation of the qPCR assay

The specificity of the endophyte-specific primer pair was verified by blasting the sequence (BLASTN search), and no other species showed 100% identity over the full query. Furthermore, the primers were tested with 10 other brown algal species. Electrophoresis on an agarose gel resulted in no visible bands for any sample except *L. elsbetiae*, suggesting a strong specificity of the primer pair (Online Resource 1).

Artificial mixtures with the same amount of host DNA and different amounts of endophyte DNA were used to test if varying amounts of *L. elsbetiae* could be detected reliably, even in low concentrations (Online Resource 2A + B). Similar quantification cycles (C_q_) were obtained with the CG primer pair. Since only small amounts of endophyte DNA were added, the total amount of DNA did not change significantly (Online Resource 2A). At the contrary, quantification of the same mixtures with the endophyte-specific primer pair (Online Resource 2B) resulted in different C_q_ values, showing that the qPCR amplification was sufficiently discriminant to detect different concentrations of total endophyte DNA over the assessed range from 0.0024 to 0.3 ng μL^−1^ total DNA.

Standard curves were drawn for both primer pairs to define the linear dynamic range of stable quantification and to compare the efficiency of amplification. As the efficiency of both primer pairs was similar (88.74% for the CG primer pair and 91.08% for the *Laminarionema* specific primer pair, Online Resource 3A + B), no efficiency correction was applied. For the primer pair CG64 and CG65, a reliable quantification was possible for cycle numbers between 18 and 29 (Online Resource 3A). C_q_ values of all samples lay within the range of this standard curve. For the LelsITS1 primer pair, the linear quantification range was between 19 and 32 cycles (Online Resource 3B). Thus, a maximal ΔC_q_ value of 14 (32–18) was set for stable quantification of *L. elsbetiae* according to the standard curves. Samples with higher C_q_ values or no endophytes were marked as “undetected”.

### Distribution of endophyte filaments along the thallus of *S. latissima*

**Fig. 1:**
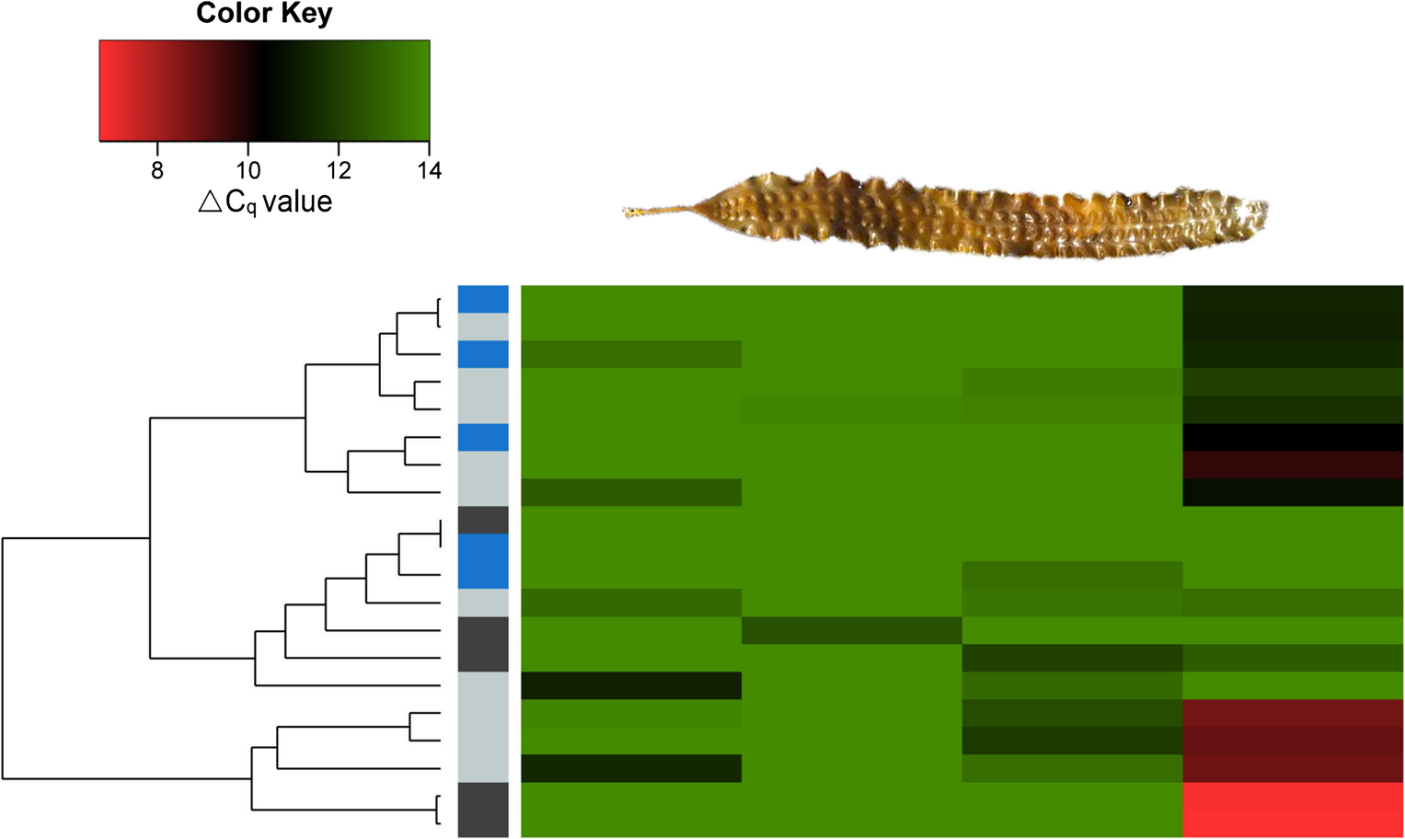
Distribution of endophyte DNA in field sporophytes of *S. latissima* (> 160 cm length) collected between March and April 2016. The small column on the left indicates the geographic origin of the sporophytes: light grey = Perharidy (Northern Brittany) *N* = 10, light blue = Locmariaquer (Southern Brittany) *N* = 5, and dark grey = bridge over the Atlantic (Western Scotland) *N* = 5. The four columns of the heat map indicate the four positions: (1) 50% of the stipe; (2) 10% of the blade length = youngest part of the blade, near meristem; (3) 50% of the blade length; and (4) 90% of the blade length = oldest part of the blade. The colours of the heat map represent ΔC_q_ values obtained by qPCR: green represents absence and red strong presence of *L. elsbetiae*

To determine the distribution of *L. elsbetiae* along the thallus of *S. latissima*, a relative infection map was established by quantifying relative infection rates at four different positions along the thallus. Endophyte filaments of *L. elsbetiae* were unequally distributed within the host, with significantly more endophyte DNA being present in the blade tip (ΔC_q_ = 10.8 ± 3.17) than in the stipe (ΔC_q_ = 13.72 ± 0.72), at 10% of the blade length (ΔC_q_ = 13.94 ± 0.29) and at 50% of the blade length (ΔC_q_ = 13.62 ± 0.63, Fig. 1, Kruskal-Wallis test, *p* ≤ 0.01, Online Resource 5). The unequal distribution along the thallus was the same in kelps from all three geographic locations. Due to this result, the samples for the following studies were taken in the blade tips of the kelps, where most endophytes were expected to be present.

**Fig. 2:**
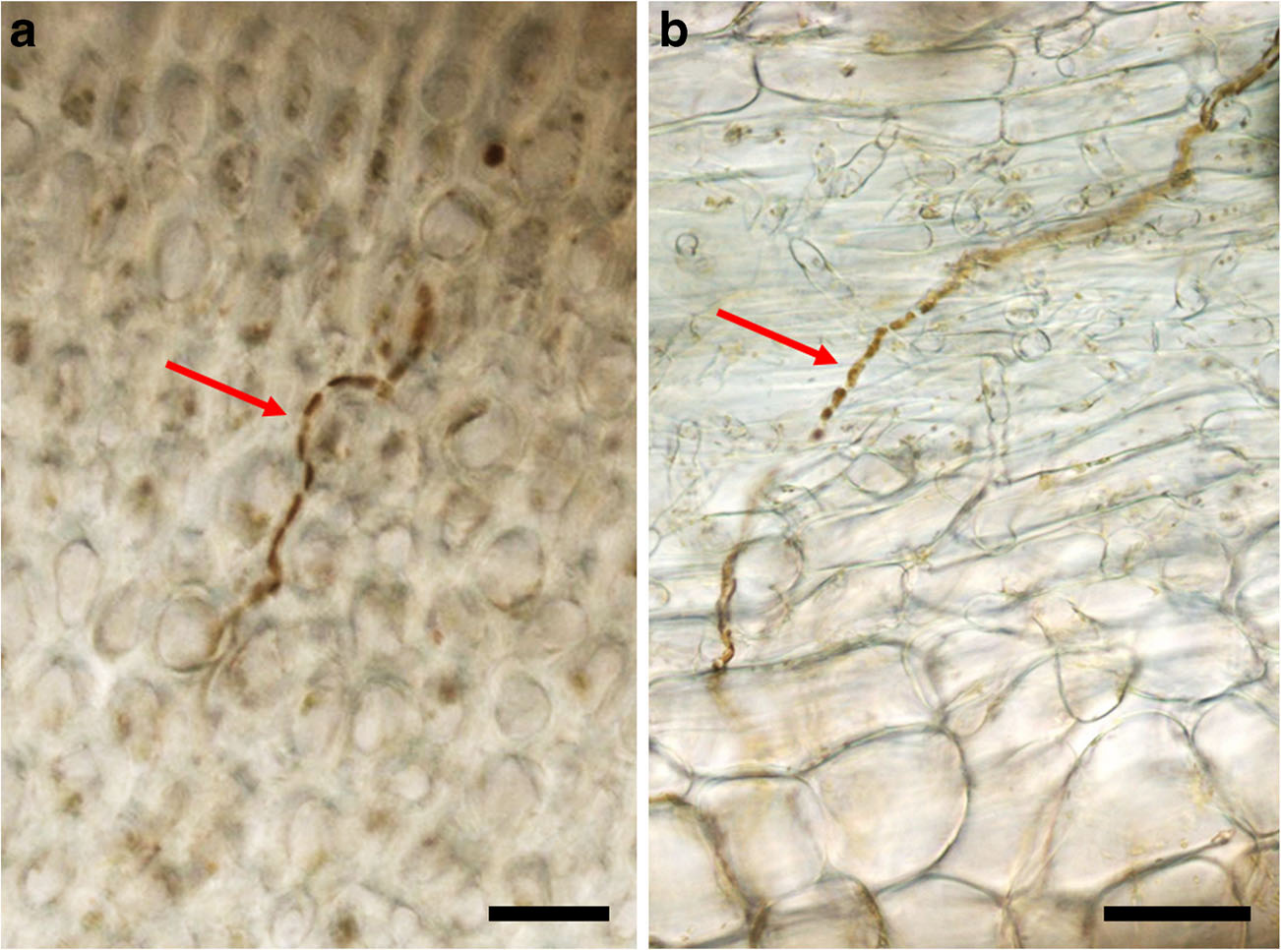
**a** A microscopic section of the stipe (position 1, 50% of the stipe length) of *S. latissima* from Northern Brittany. **b** A microscopic section of the blade tip (positions 4, 90% of the blade length) of *S. latissima* from Northern Brittany. Red arrows indicate endophytic filaments, and the scale bar presents 25 μm

The presence of filamentous brown algae at the four positions in the same *Saccharina* sporophytes was also examined in microscopic sections (Fig. 2). Eighty percent of the thalli from Northern Brittany and Western Scotland and 60% of the thalli from Southern Brittany contained filamentous algae in the blade tips (Fig. 2b). Seventy percent and 20% of the stipe sections of sporophytes from Northern Brittany and Southern Brittany, respectively, contained endophytic filaments (Fig. 2a) while no filament was detected in the stipe sections of the *S. latissima* sporophytes from Western Scotland. In all examined sections, no endophytic filaments were visible in the intermediate sections (positions 2 and 3 in Fig. 1).

### Infection rates in young kelps

**Fig. 3:**
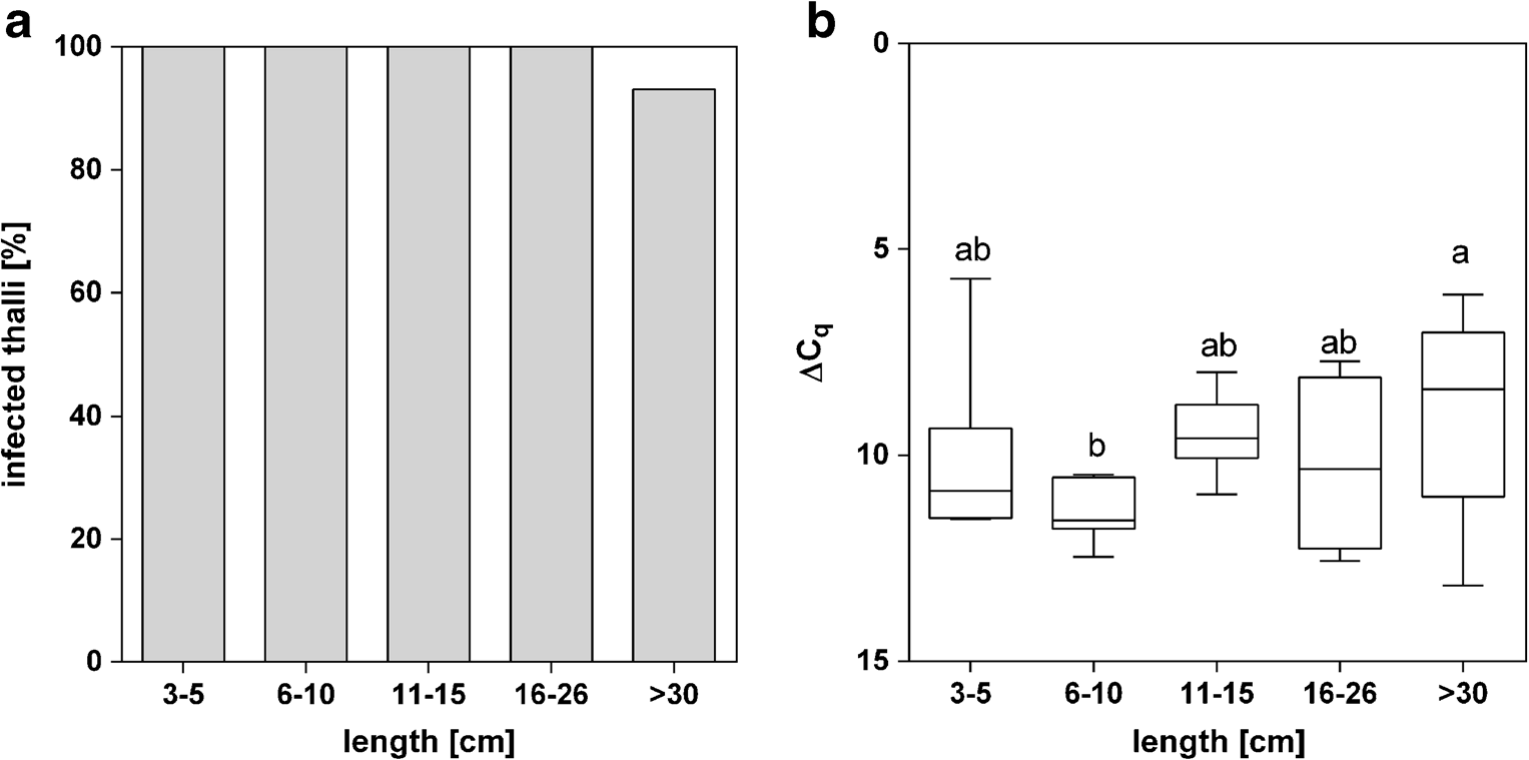
**a** Percentage of *S. latissima* sporophytes with different thallus lengths infected with *L. elsbetiae*. **b** ΔC_q_ values obtained by qPCR represent the relative amount of *L. elsbetiae* in *S. latissima* individuals of different thallus lengths obtained from punch-outs of blade tips collected in March 2017 (3–5 cm, *N* = 6; 6–10 cm, *N* = 7; 11–15 cm, *N* = 8; 16–26 cm, *N* = 4; > 30 cm, *N* = 30). Whiskers indicate the smallest and largest values, and letters indicate statistically significant differences (one-way ANOVA)

The occurrence of the endophyte infection in the field was investigated by determining relative infection rates of young *S. latissima* sporophytes. One hundred percent of the young *S. latissima* sporophytes collected in Northern Brittany contained DNA of *L. elsbetiae*, compared to 93% of infected thalli in old (> 30 cm) sporophytes collected at the same time (Fig. 3a). When comparing the relative infection rates, no significant difference appeared in the ΔC_q_ between sporophytes of all lengths (ΔC_q_ = 9.82 ± 0.6, Fig. 3b) except for the samples with a thallus length from 6 to 10 cm where infection rates were slightly lower (ΔC_q_ = 11.43 ± 0.7, Fig. 3b).

### Natural infection of laboratory-grown samples in a seaweed farm

**Fig. 4:**
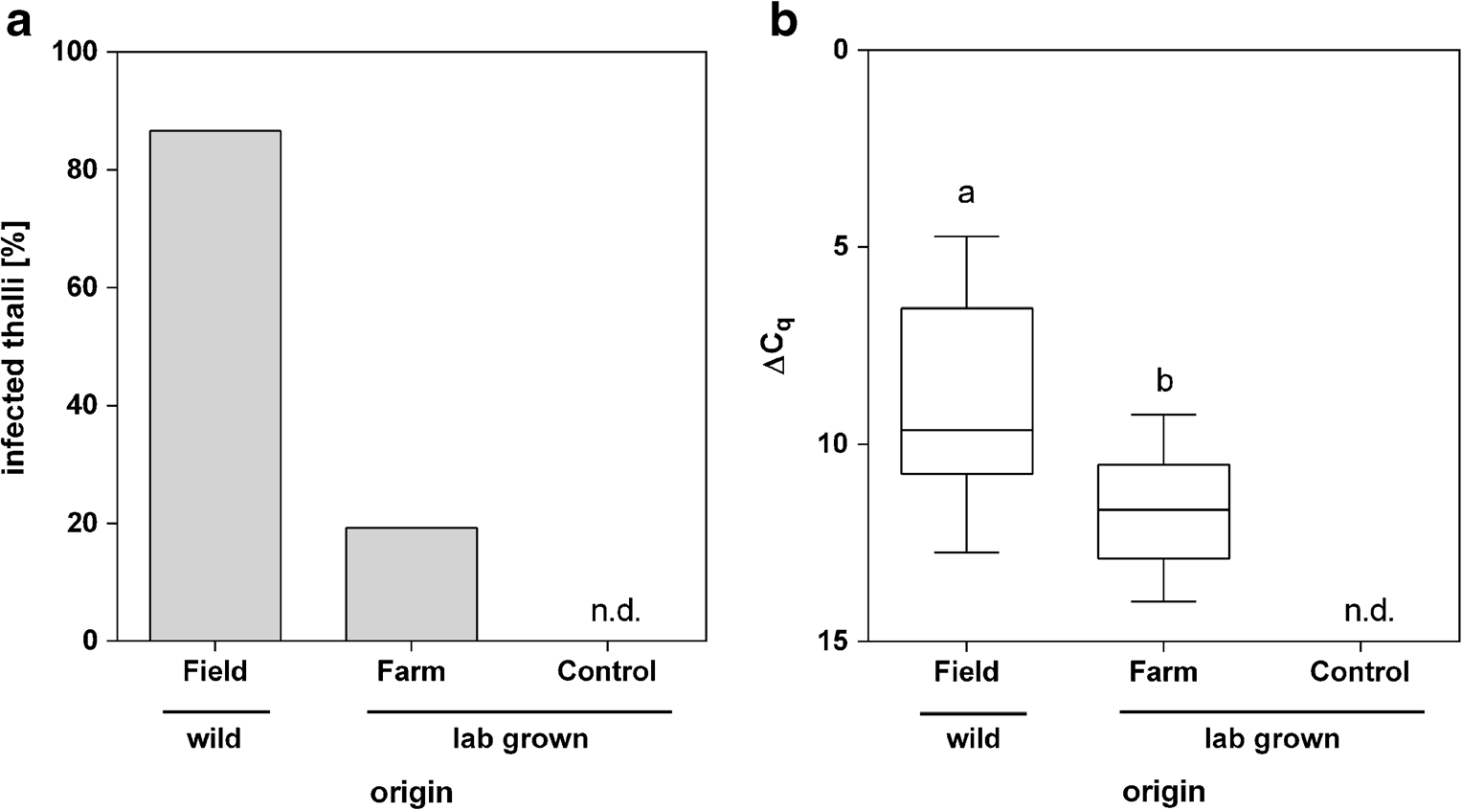
**a** Percentage of *S. latissima* sporophytes from different origins infected with *L. elsbetiae*. **b** ΔC_q_ values obtained by qPCR represent the relative amount of *L. elsbetiae* in *S. latissima* obtained in October 2016 from a wild population in Northern Brittany (*N* = 30) and from laboratory-grown samples transferred to a seaweed farm (*N* = 57) and kept under laboratory conditions (*N* = 27). Whiskers indicate the smallest and largest values, and letters indicate statistically significant differences (one-way ANOVA), n.d. = no *L. elsbetiae* detected by qPCR

The course of natural infection of *S. latissima* with *L. elsbetiae* was further explored by an experimental set-up where laboratory-grown samples were transferred to a seaweed farm for 6 months and infection rates were compared to samples from a wild population. The number of thalli infected with *L. elsbetiae* was more than four times higher in wild samples (87% of infected thalli) than in the samples grown in the seaweed farm in close vicinity to the wild population (19%, Fig. 4a). No endophytes were detected in the laboratory controls (Fig. 4a). While the laboratory-grown samples in the seaweed farm were heavily covered with epiphytes (data not shown), the qPCR revealed significantly lower infection rates by the endophyte *L. elsbetiae* (ΔC_q_ = 11.81 ± 1.4) as compared to wild samples (ΔC_q_ = 8.99 ± 2.5) (one-way ANOVA, *p* ≤ 0.01, Fig. 4b, Online Resource 6).

### Seasonal variation of relative infection rates

**Fig. 5:**
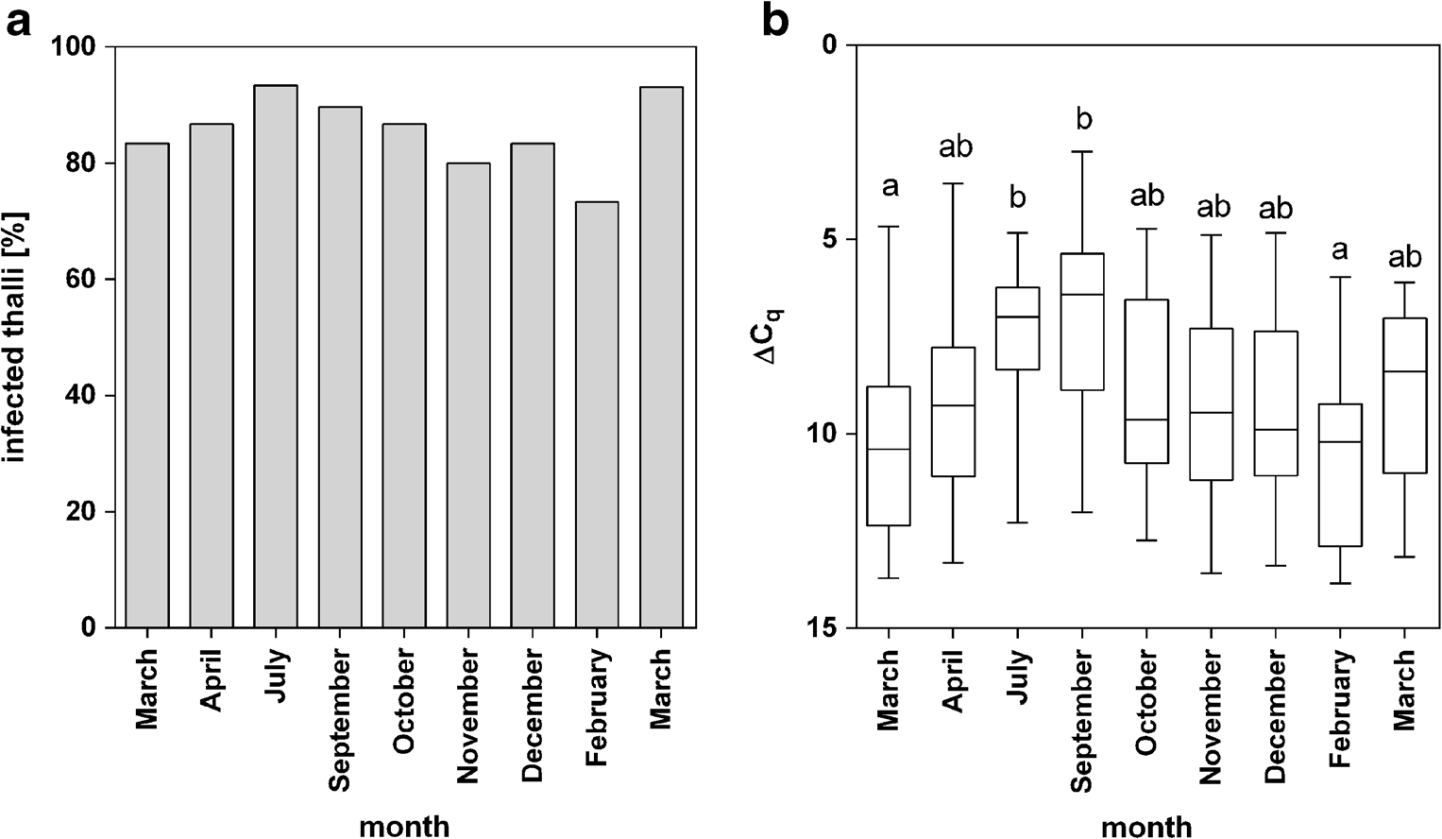
**a** Percentage of *S. latissima* sporophytes collected in different months infected with *L. elsbetiae* in a population of *S. latissima* at Perharidy (Northern Brittany). **b** Seasonal changes in the relative amount of *L. elsbetiae* in a population of *S. latissima* at Perharidy (Northern Brittany) represented by ΔC_q_ values obtained from punch-outs of blade tips of 30 individuals per sampling. Whiskers indicate the smallest and largest values, and letters indicate statistically significant differences (one-way ANOVA)

To examine the seasonal variation of infection in a natural population, regular samplings took place in Northern Brittany from March 2016 to March 2017. The endophyte prevalence in the *S. latissima* population ranged between 73 and 93% with the lowest number of infected kelps detected in February 2017 (73%) and most kelps infected in July 2016 and March 2017 (93%, Fig. 5a). The relative amount of *L. elsbetiae* filaments in infected thalli also increased during spring and was significantly higher between July and September (ΔC_q_ = 7.38 ± 1.8 and 7.06 ± 2.4, respectively) than during the rest of the year (one-way ANOVA, *p* ≤ 0.01, Fig. 5b, Online Resource 6). Infection rates decreased in October, reaching the lowest value in February (ΔC_q_ = 10.75 ± 2.1) and increasing again in March (Fig. 5b).

### Geographic variation of relative infection rates

**Fig. 6:**
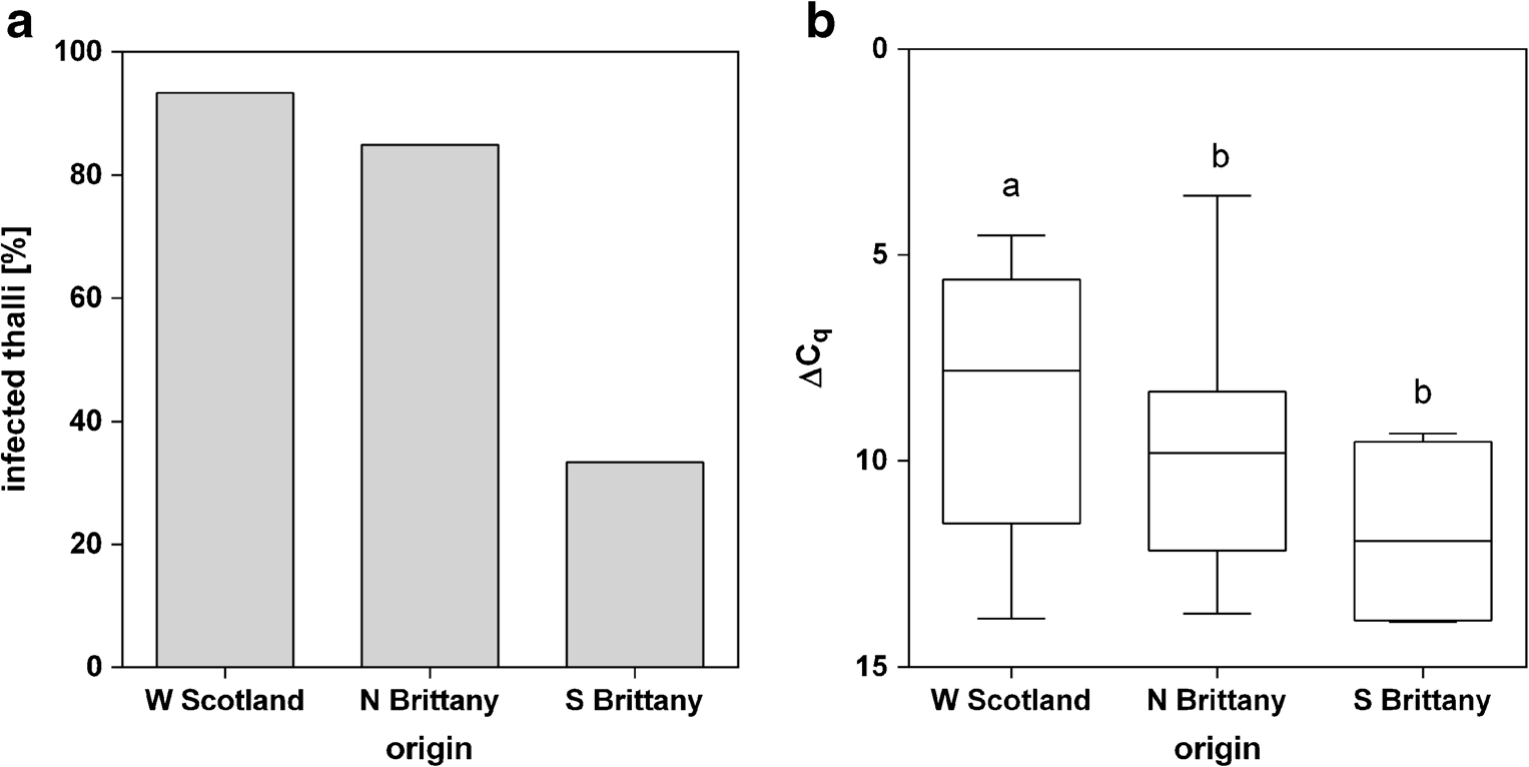
**a** Percentage of *S. latissima* sporophytes from different geographic origins infected with *L. elsbetiae*. **b** ΔC_q_ values obtained by qPCR represent the relative amount of *L. elsbetiae* in *S. latissima*, obtained from punch-outs of blade tips of individuals from Western Scotland (*N* = 30), Northern Brittany (*N* = 60) and Southern Brittany (*N* = 12), collected in March and/or April 2016. Whiskers indicate the smallest and largest values, and letters indicate statistically significant differences (one-way ANOVA)

Geographic variation of relative infection rates of *L. elsbetiae* in *S. latissima* sporophytes was determined by samplings in three different populations in Southern Brittany, Northern Brittany and Western Scotland during March and April 2016. While 85 and 93% of the sporophytes collected in Northern Brittany and Western Scotland, respectively, were infected with the endophyte, only 33% of sporophytes from Southern Brittany contained detectable amounts of endophytic filaments in their blade tips (Fig. 6a). Moreover, the relative infection rate by *L. elsbetiae* was shown to differ significantly between Western Scotland and Brittany (one-way ANOVA, *p* = 0.01, Fig. 6b, Online Resource 6). Kelps collected in Southern Brittany (Locmariaquer) contained significantly less *L. elsbetiae* (ΔC_q_ = 11.78 ± 2.4) than the ones from Northern Brittany (Perharidy, ΔC_q_ = 9.93 ± 2.3). The sporophyte samples collected at the Bridge over the Atlantic (Western Scotland) were most heavily infected (ΔC_q_ = 8.39 ± 3, Fig. 6b).

### Host specificity

**Fig. 7:**
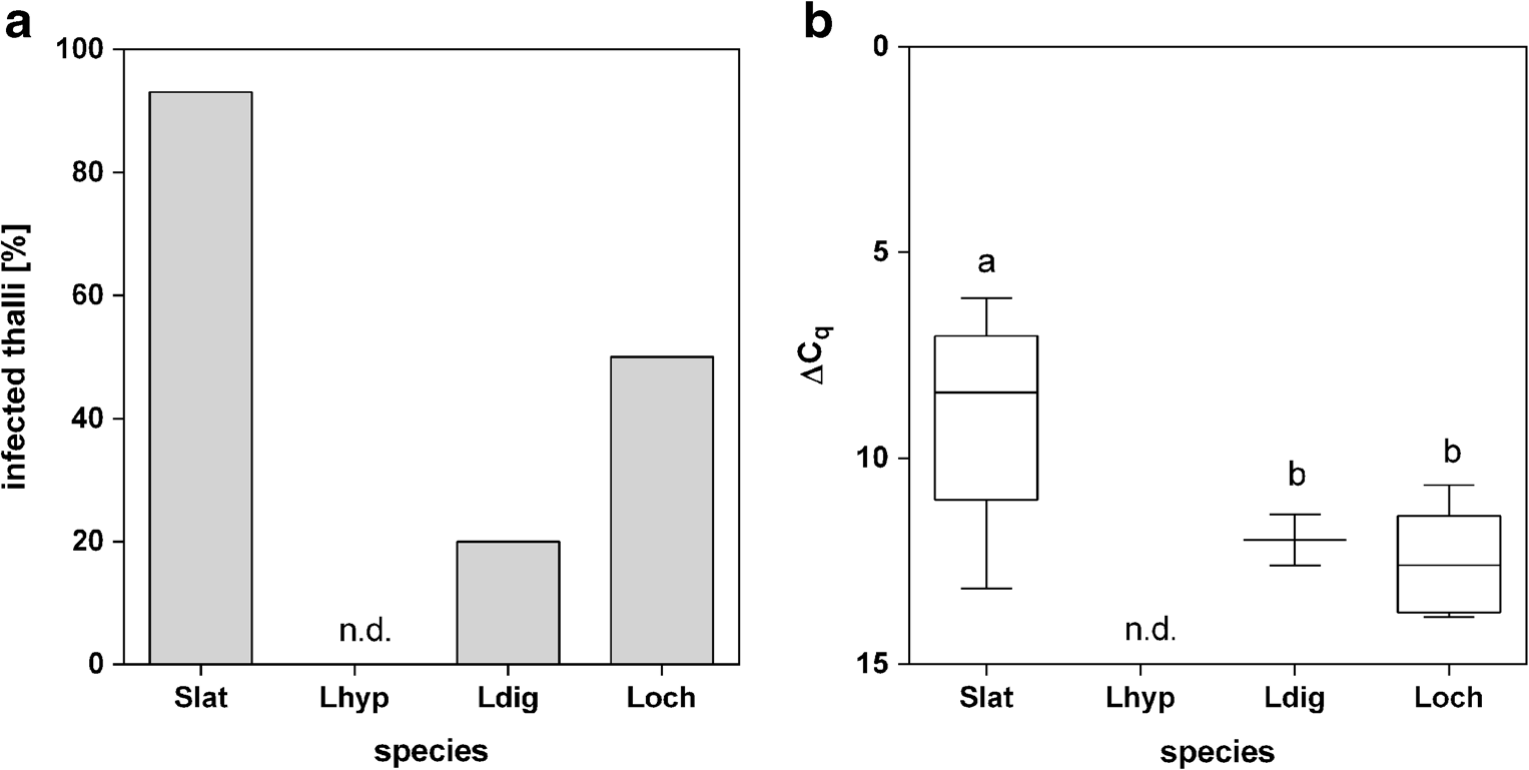
**a** Percentage of sporophytes from different species infected with *L. elsbetiae*. **b** ΔC_q_ values obtained by qPCR represent the relative amount of *L. elsbetiae* in *S. latissima* (Slat, *N* = 30), *L. hyperborea* (Lhyp, *N* = 10), *L. digitata* (Ldig, *N* = 10) and *L. ochroleuca* (Loch, *N* = 10), obtained from punch-outs of blade tips, collected in March 2017. Whiskers indicate the smallest and largest values, and letters indicate statistically significant differences (one-way ANOVA), n.d, no *L. elsbetiae* detected by qPCR

To explore the host specificity of *L. elsbetiae*, endophyte prevalence and infection rates of *S. latissima* were compared to infection rates of the adjacent kelp species *L. hyperborea*, *L. digitata* and *L. ochroleuca*. While 93% of the *S. latissima* sporophytes were infected with *L. elsbetiae*, endophyte DNA was only detected in 20 and 50% of the *L. digitata* and *L. ochroleuca* individuals, respectively, collected at the same location and the same time (Fig. 7a). *Laminarionema elsbetiae* was not detected in DNA from any sample of *L. hyperborea*. Additionally, infection rates in *S. latissima* sporophytes were significantly higher (ΔC_q_ = 8.97 ± 2) than in *L. digitata* and *L. ochroleuca* individuals adjacent to the *Saccharina* population (ΔC_q_ = 11.98 ± 0.9 and 12.58 ± 1.3, respectively, one-way ANOVA, *p* ≤ 0.01, Fig. 7b, Online Resource 6).

DNA of *L. elsbetiae* was also specifically amplified in the seawater samples collected at three locations in close vicinity to *Saccharina* sporophytes (Online Resource 4).

## Discussion

### A specific and reliable qPCR approach for epidemiological studies

In our study, we detected a high prevalence of *L. elsbetiae* in *S. latissima* with up to 100% of infected individuals in Northern Brittany. This is in consistence with previous epidemiological studies about filamentous endophytes in brown algae in the Atlantic and Pacific Ocean (Andrews 1977; Lein et al. 1991; Peters and Schaffelke 1996; Ellertsdóttir and Peters 1997; Peters 2003). Until now, epidemiological studies were based on different methodological approaches, rendering them difficult to compare. Andrews (1977) determined an infection rate of 20% by quantifying galls on *S. latissima* presumably caused by a filamentous brown algal endophyte. However, the presence of endophyte filaments is not always directly connected to the occurrence of disease symptoms (Ellertsdóttir and Peters 1997; Gauna et al. 2009b) and indeed most of the sporophytes infected with *L. elsbetiae* sampled in our study did not show any disease symptoms. Thus, an epidemiological survey based on the occurrence of symptoms could lead to an underestimation if endophytes do not cause identifiable changes in the host. Other studies were based on counting endophyte filaments in microscopic sections (Lein et al. 1991; Gauna et al. 2009a). While this method provides valuable information about the presence of filamentous endophytes, a precise quantification by visual scoring and the subsequent statistical analysis are difficult. Furthermore, filamentous endophytes are difficult to identify in microscopic sections as species are morphologically little differentiated (Eggert et al. 2010). For a trustworthy identification, the endophyte needs to be isolated and cultivated in a time-consuming process (Ellertsdóttir and Peters 1997; Peters 2003; Amsler et al. 2009).

The evaluation of our qPCR assay confirmed that this new approach is suitable not only for a relative quantification of the prevalence and the severity of infection but also for a specific, rapid and sensitive identification of *L. elsbetiae*. A possible concern might be that the *L. elsbetiae*-specific primer pair could amplify other so far unknown species. However, up to now, only one species of the genus *Laminarionema* is described and the related genera *Laminariocolax* and *Microspongium* which also contain filamentous endophytes (Peters and Burkhardt 1998; Peters 2003) are not targeted by the *L. elsbetiae*-specific primer pair (Online Resource 1). The results obtained using the qPCR assay correlate with the detection of endophytic filaments by microscopy in highly infected parts. Moreover, low amounts of endophyte DNA could be detected by qPCR in parts of the blade where no filaments where visible in the microscopic sections showing that the qPCR assay is a more sensible tool than microscopy.

### Early occurrence of the infection in nature

Since it has been reported for other host-endophyte pairs that endophytic filaments can be distributed unequally within the host (Amsler et al. 2009; Gauna et al. 2009a), we investigated the distribution of endophytes along the host thallus and confirmed that most endophytes were located in the blade tips of *S. latissima*. This stresses the importance of careful planning of samplings for epidemiological studies, as the infection rates may differ significantly depending on where exactly on the thallus samples are taken. The meristematic tissue of kelps lies at the junction between stipe and blade; the blade tip is therefore the oldest part of the sporophyte (Wilkinson 1995). The concentration of endophyte filaments in the blade tip could indicate that hosts are infected very early in their life, and the endophyte subsequently stays in the same tissue while this part grows further away from the meristem. To test this hypothesis, we collected young kelps of different lengths in order to compare the infection rates with the old sporophytes*. Laminarionema elsbetiae* was detected in all of the sampled kelps, even the very young ones (> 5 cm), suggesting an early infection of the kelp. Furthermore, kelps from a seaweed farm, which had been grown in lab conditions for 10 weeks, were significantly less infected than wild kelps although the farm and population were in vicinity to each other (4 km distance), i.e. exposed to similar environmental conditions. These kelps were kept in the laboratory during their early life, and once they were taken out to the sea, the cell walls might have already changed enough to make it more difficult for the endophyte to enter (Apt 1988a). Similarly, in the case of the closely related pacific kelp species *Saccharina japonica*, only young specimen could be infected by the filamentous brown algal endophyte *Streblonema* sp., while the filaments of the endophyte could not penetrate the tissue of mature kelps, unless it presented a wounding site (Apt 1988a). Previous experimental infection of *S. latissima* with *Laminarionema* used very young host sporophytes (< 10 mm in length), which were readily infected (Heesch and Peters 1999). Overall, our results suggest that *S. latissima* is infected with *L. elsbetiae* while it is still very young and keeping *Saccharina* cultures under controlled conditions for a certain amount of time could reduce infection rates of cultivated *S. latissima* with *L. elsbetiae*.

### Variation of infection rates

The severity of infection differed not only along the thallus but also depended on seasonal and geographic location. Infection rates within the *Saccharina* population in Northern Brittany were significantly higher in summer as compared to the rest of the year. This is in agreement with observations on endophytic infections in *S. latissima* and two other kelp species on Helgoland (Ellertsdóttir and Peters 1997) and in *S. latissima* in the Pacific Northwest (Andrews 1977). However, as seasonal samplings were only conducted in Northern Brittany, additional samplings at other locations are necessary to confirm a general pattern of seasonal variation. Kelps may lose distal parts of their blade in winter, thereby shedding infected tissue (Ellertsdóttir and Peters 1997). Furthermore, it is possible that growth rates of the endophyte benefit from higher summer temperatures. Seasonal variation of infection rates could also be connected to the life cycle of *L. elsbetiae*. The endophyte spreads between hosts via zoospores that penetrate the host tissue (Heesch and Peters 1999) and on Helgoland fertile structures in *L. elsbetiae* were found only during spring (Peters and Ellertsdóttir 1996). As we were able to detect *L. elsbetiae* DNA in seawater samples taken around a *S. latissima* population in spring, it is likely that spores of the endophyte were present in the seawater during this time, spreading to infect new hosts.

Significant differences were found between kelp populations from France and Scotland both, in endophyte prevalence and infection rates, increasing from Southern Brittany to Western Scotland. Seawater temperature is decreasing along a latitudinal gradient from 14.1 °C in Locmariaquer (Southern Brittany) to 12.4 °C in Perharidy (Northern Brittany) and 9.5 °C of average annual sea surface temperature in Oban (Western Scotland, data provided by the National Oceanic and Atmospheric Administration). However, temperature is not the only factor that discriminates the three populations. The *Saccharina* populations in Western Scotland and Northern Brittany are also denser than the one in Southern Brittany, which lies near the distribution limit of *S. latissima* and host density plays an important role in spreading infective agents like spores (Clay 1990). Furthermore, the different examined populations are exposed to different strengths of currents. Ellertsdóttir and Peters (1997) found that endophyte prevalence was higher at more wave-exposed sites. Water depth is another factor that has a significant impact on endophyte distribution, with stronger disease symptoms in shallow water than in deep water (Schaffelke et al. 1996; Ellertsdóttir and Peters 1997) either by reducing the host fitness under higher UV radiation or by favouring endophyte growth rates due to higher PAR (Schaffelke et al. 1996). Since environmental factors seem to have a significant impact on the host-endophyte relationship, experiments under controlled laboratory conditions are necessary to examine the effect of single environmental factors on the interaction between *S. latissima* and *L. elsbetiae*.

### Host specificity of *L. elsbetiae*

Both the number of infected thalli and the severity of infection with *L. elsbetiae* were significantly higher in *S. latissima* than in other kelps collected in the vicinity. Similar to results obtained by microscopic observations and subsequent isolation from kelps on Helgoland (Ellertsdóttir and Peters 1997), we detected *L. elsbetiae* also in 20% of *L. digitata*. Additionally, DNA of *L. elsbetiae* was found in 50% of *L. ochroleuca* sporophytes, but not in *L. hyperborea*, whereas *L. elsbetiae* spores were likely to be present in the surrounding seawater.

*Laminarionema elsbetiae* was first described based on isolates from *S. japonica* in Japan, where it was not found infecting any other kelp species in close vicinity, pointing out a high specificity of the infection (Kawai and Tokuyama 1995). Electron microscopy of the infection process suggested that *L. elsbetiae* enters the tissue of *S. latissima* by enzymatic dissolution of the cell wall (Heesch and Peters 1999), but so far, it is still unclear what exactly defines the ability of the endophyte to infect certain hosts. Chemical differences in kelp cell wall compositions—for example in the content of celluloses, hemicelluloses and alginates (Siegel and Siegel 1973)—could play an important part in the host specificity of algal endophytes. As the cell wall composition of brown algae is known to vary based on environmental conditions (Rosell and Srivastava 1984; Adams et al. 2011; Deniaud-Bouët et al. 2014), these differences could also contribute to geographic and seasonal variations in the endophyte prevalence. Furthermore, host specificity might be based on different kelp species having specific defence reactions. The oxidative burst upon elicitation with oligoguluronates, an early defence response, differs amongst several members of the Laminariales (Küpper et al. 2002). Finally, the ability of an endophyte to infect a host is also strongly linked to the life cycles of both, the host and the endophyte. As our results suggest that kelps are infected at a very young age, host specificity might be coupled to the occurrence of young sporophytes of different species in the field and the synchronisation of spore release from *L. elsbetiae*. Fertility periods and subsequently the appearance of young sporophytes are known to be variable within the Laminariales (Bartsch et al. 2008), but further studies on the life cycle of *L. elsbetiae* are necessary to better understand these relationships.

Overall, the consistency in results of our approach with microscopic observation and previous epidemiological studies based on other methods confirm the reliability of our qPCR assay. This efficient tool is well adapted for routine application and processing of large sample numbers for epidemiological studies on infections of *S. latissima* with *L. elsbetiae*. Moreover, the approach could be easily transferred to other host-endophyte pairs by designing specific primers and therefore be applied to extensive studies on kelp-endophyte interactions.

## Electronic supplementary material

ESM 1 (DOCX 320 kb).

## References

[CR1] Adams JM, Gallagher JA, Donnison IS (2009). Fermentation study on *Saccharina latissima* for bioethanol production considering variable pre-treatments. J Appl Phycol.

[CR2] Adams JMM, Ross AB, Anastasakis K, Hodgson EM, Gallagher JA, Jones JM, Donnison LS (2011). Seasonal variation in the chemical composition of the bioenergy feedstock *Laminaria digitata* for thermochemical conversion. Bioresour Technol.

[CR3] Amsler CD, Amsler MO, McClintock JB, Baker BJ (2009). Filamentous algal endophytes in macrophytic Antarctic algae: prevalence in hosts and palatability to mesoherbivores. Phycologia.

[CR4] Andrews JH (1977). Observations on the pathology of seaweeds in the Pacific Northwest. Can J Bot.

[CR5] Apt KE (1988). Etiology and development of hyperplasia induced by *Streblonema* sp. (Phaeophyta) on members of the Laminariales (Phaeophyta). J Phycol.

[CR6] Apt KE (1988). Galls and tumor-like growths on marine macroalgae. Dis Aquat Org.

[CR7] Apt KE, Clendennen SK, Powers DA, Grossman AR (1995). The gene family encoding the fucoxanthin chlorophyll proteins from the brown alga *Macrocystis pyrifera*. Mol Gen Genet.

[CR8] Bartsch I, Wiencke C, Bischof K, Buchholz CM, Buck BH, Eggert A, Feuerpfeil P, Hanelt D, Jacobsen S, Karez R, Karsten U, Molis M, Roleda MY, Schubert H, Schumann R, Valentin K, Weinberger F, Wiese J (2008). The genus *Laminaria sensu lato*: recent insights and developments. Eur J Phycol.

[CR9] Brouwer M, Lievens B, Van Hemelrijck W, Van den Ackerveken G, Cammue BP, Thomma BP (2003). Quantification of disease progression of several microbial pathogens on *Arabidopsis thaliana* using real-time fluorescence PCR. FEMS Microbiol Lett.

[CR10] Bustin SA, Benes V, Garson JA, Hellemans J, Huggett J, Kubista M, Mueller R, Nolan T, Pfaffl MW, Shipley GL, Vandesompele J, Wittwer CT (2009). The MIQE guidelines: Minumum information for publication of quantitative real-time PCR experiments. Clin Chem.

[CR11] Chen J (2004) Cultured aquatic species information programme. *Laminaria japonica*. In: FAO Fisheries and Aquaculture Department [online]. Rome. Updated 1 January 2004. [Cited 18 May 2017]

[CR12] Clay K, Grace JB, Tilman D (1990). The impact of parasitic and mutualistic fungi on competitive interactions among plants. Perspectives on plant competition.

[CR13] Deniaud-Bouët E, Kervarec N, Michel G, Tonon T, Kloareg B, Hervé C (2014). Chemical and enzymatic fractionation of cell walls from Fucales: insights into the structure of the extracellular matrix of brown algae. Ann Bot.

[CR14] Eggert A, Peters AF, Küpper FC, Seckbach J, Einav R, Israel A (2010). Potential impact of climate change on endophyte infections in kelp sporophytes. Seaweeds and their role in globally changing environment.

[CR15] Ellertsdóttir E, Peters AF (1997). High prevalence of infection by endophytic brown algae in populations of *Laminaria* spp. (Phaeophyceae). Mar Ecol Prog Ser.

[CR16] Gachon C, Mingam A, Charrier B (2004). Real-time PCR: what relevance to plant studies?. J Exp Bot.

[CR17] Gachon CMM, Strittmatter M, Mueller DG, Kleinteich J, Kuepper FC (2009). Detection of differential host susceptibility to the marine oomycete pathogen *Eurychasma dicksonii* by real-time PCR: not all algae are equal. Appl Environ Microbiol.

[CR18] Gauna C, Parodi ER, Caceres E (2009). The occurrence of *Laminarionema elsbetiae* (Phaeophyta) on *Rhodymenia pseudopalmata* (Rhodophyta) from the Patagonian coasts of Argentina: characteristics of the relationships in natural and experimental infections and morphology of the epi-endophyte in unialgal free cultures. Algae.

[CR19] Gauna MC, Parodi ER, Cáceres EJ (2009). Epi-endophytic symbiosis between *Laminariocolax aecidioides* (Ectocarpales, Phaeophyceae) and *Undaria pinnatifida* (Laminariales, Phaeophyceae) growing on Argentinian coasts. J Appl Phycol.

[CR20] Heesch S, Peters AF (1999). Scanning electron microscopy observation of host entry by two brown algae endophytic in *Laminaria saccharina* (Laminariales, Phaeophyceae). Phycol Res.

[CR21] Kawai H, Tokuyama M (1995). *Laminarionema elsbetiae* gen. et sp. nov. (Ectocarpales, Phaeophyceae), a new endophyte in *Laminaria* sporophytes. Phycol Res.

[CR22] Küpper FC, Müller DG, Peters AF, Kloareg B, Potin P (2002). Oligoalginate recognition and oxidative burst play a key role in natural and induced resistance of sporophytes of Laminariales. J Chem Ecol.

[CR23] Lein TE, Sjotun K, Wakili S (1991). Mass-occurrence of a brown filamentous endophyte in the lamina of the kelp *Laminaria hyperborea* (Gunnerus) Foslie along the southwestern coast of Norway. Sarsia.

[CR24] Østgaard K, Indergaard M, Markussen S, Knutsen SH, Jensen A (1993). Carbohydrate degradation and methane production during fermentation of *Laminaria saccharina* (Laminariales, Phaeophyceae). J Appl Phycol.

[CR25] Peters AF (1991). Field and culture studies of *Streblonema macrocystis* sp. nov. (Ectocapales, Phaeophyceae) from Chile, a sexual endophyte of giant kelp. Phycologia.

[CR26] Peters AF, Chapman ARO, Anderson RJ, Vreeland V (2003). Molecular identification, distribution and taxonomy of brown algal endophytes, with emphasis on species from Antarctica. Proceedings of the 17th International Seaweed Symposium.

[CR27] Peters AF, Burkhardt E (1998). Systematic position of the kelp endophyte *Laminarionema elsbetiae* (Ectocarpales *sensu lato*, Phaeophyceae) inferred from nuclear ribosomal DNA. Phycologia.

[CR28] Peters AF, Ellertsdóttir E (1996). New record of the kelp endophyte *Laminarionema elsbetiae* (Phaeophyceae, Ectocarpales) at Helgoland and its life history in culture. Nova Hedwigia.

[CR29] Peters AF, Schaffelke B (1996). *Streblonema* (Ectocarpales, Phaeophyceae) infection in the kelp *Laminaria saccharina* (Laminariales, Phaeophyceae) in the western Baltic. Hydrobiologia.

[CR30] Pfaffl MW (2004) Relative quantification. In: Tevfik Dorak M (ed) Real-time PCR. Taylor & Francis Group, New York, pp 63–82

[CR31] Potin P, Bouarab K, Salaün JP, Pohnert G, Kloareg B (2002). Biotic interactions of marine algae. Curr Opin Plant Biol.

[CR32] Provasoli L (1968) Media and prospects for the cultivation of marine algae. In: Watanabe A (ed) Cultures and collections of algae. Japanese Society of Plant Physiologists, Tokyo, pp 63–75

[CR33] Rosell KG, Srivastava LM (1984). Seasonal variation in the chemical constituents of the brown algae *Macrocystis integrifolia* and *Nereocystic luetkeana*. Can J Bot.

[CR34] Schaffelke B, Peters AF, Reusch T (1996). Factors influencing depth distribution of soft bottom *Laminaria saccharina* (L.) Lamour in Kiel Fjord, Baltic Sea. Hydrobiologia.

[CR35] Siegel BZ, Siegel SM (1973). The chemical composition of algal cell walls. Crit Rev Microbiol.

[CR36] Thomas D, Beltrán J, Flores V, Contreras L, Bollmann E, Correa JA (2009). *Laminariocolax* sp. (Phaeophyceae) associated with gall developments in *Lessonia nigrescens* (Phaeophyceae). J Phycol.

[CR37] Wilkinson M (1995) Information review on the impact of kelp harvesting. Scottish Natural Heritage Review, No. 34

[CR38] Wu C, Chen D, Li J, Jiajun L (1983) On the diseases of cultivated *Laminaria japonica*. In Research Report of Academia Sinica Institute of Oceanology Qingdao 763:211–221

[CR39] Yoshida T, Akiyama K (1979) *Streblonema* (Phaeophyceae) infection in the frond of cultivated *Undaria* (Phaeophyceae). In: Proc. 9th Int. Seaweed Symp. Science Press, Santa Barbara, pp 213–223

