# Supplementary material for: qPCR-based relative quantification of the brown algal endophyte Laminarionema elsbetiae in Saccharina latissima: variation and dynamics of host—endophyte interactions
Source: J Appl Phycol. 2017 Dec 19;30(5):2901–11. doi: 10.1007/s10811-017-1367-0 (PMC6208874; doi:10.1007/s10811-017-1367-0)
Supplement: Supplementary file 1 — (DOCX 320 kb). [file 10811_2017_1367_MOESM1_ESM.docx]

**Supplementary material**

**
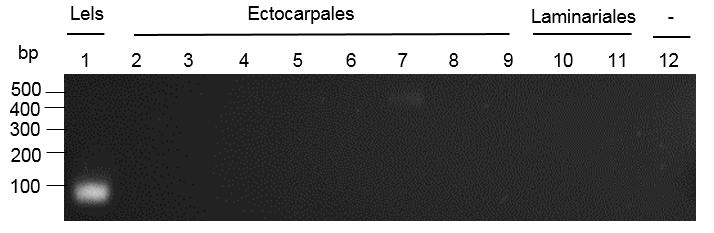
Online Resource 1** Agarose gel (2.5%) of qPCR products using the LelsITS1F2 and LelsITS1R2 primer pair. 1 = *Laminarionema elsbetiae*, 2 = *Ectocarpus* strain Ec02 F, 3 = *Ectocarpus fasciculatus*, 4 = *Microspongium tenuissimum*, 5 = *Laminariocolax aecidioides*, 6 = *Laminariocolax tomentosoides*, 7 = *Saccharina latissima*, 8 = *Laminaria digitata*, 9 = *Feldmannia mitchelliae*, 10 = *Hincksia hincksiae*, 11 = *Hecatonema maculans*, 12 = neg. control. (autoclaved milliQ H_2_O) Weight marker: SmartLadder SF (Eurogentec, Belgium)


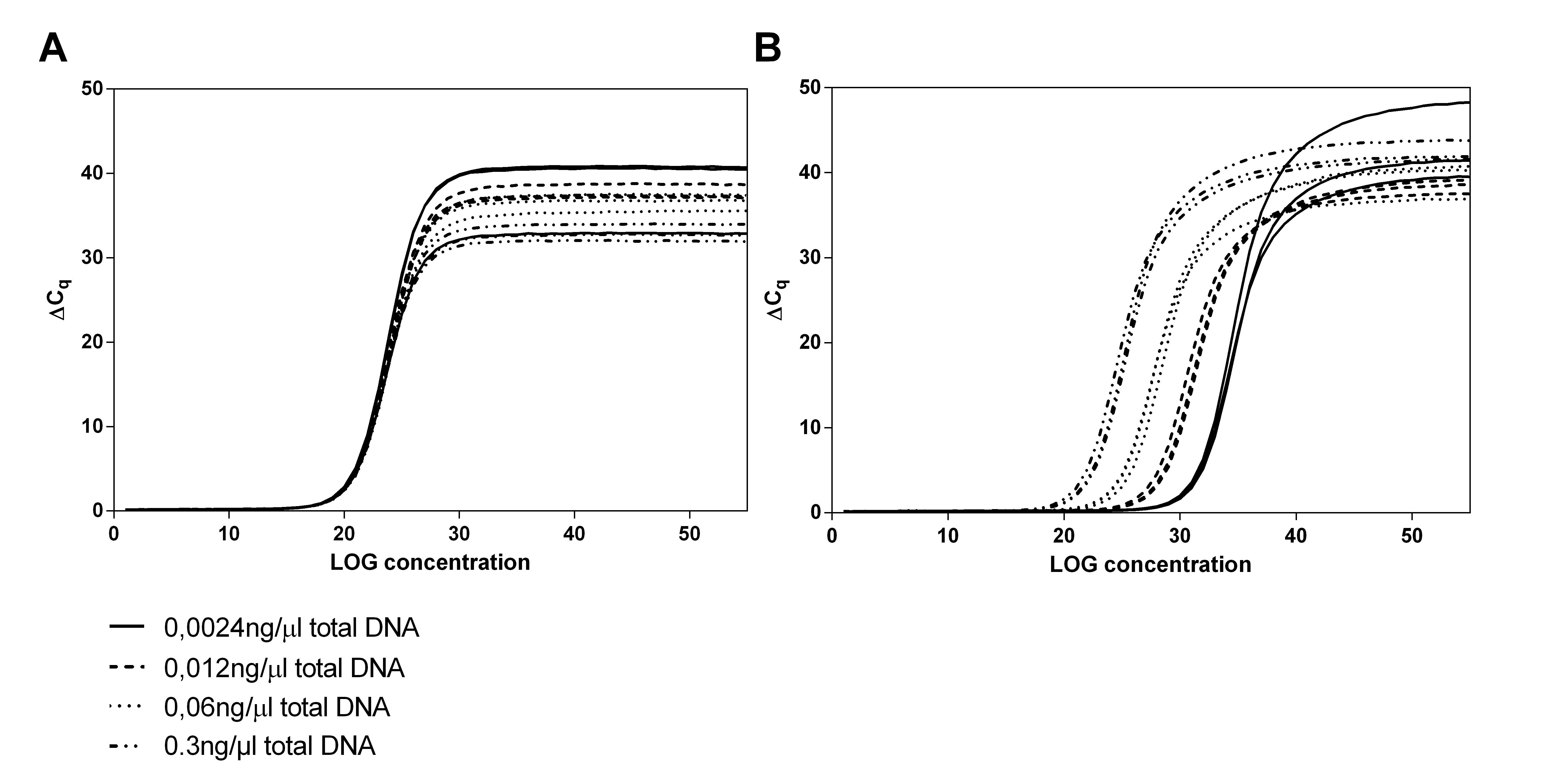


**Online Resource 2** Artificial mix of 1ng host DNA with different amounts of endophyte DNA run in triplicates: A. CG Primer pair. B. LelsITS1 primer pair. Patterns of the curve show the different amounts of endophyte DNA in the mix


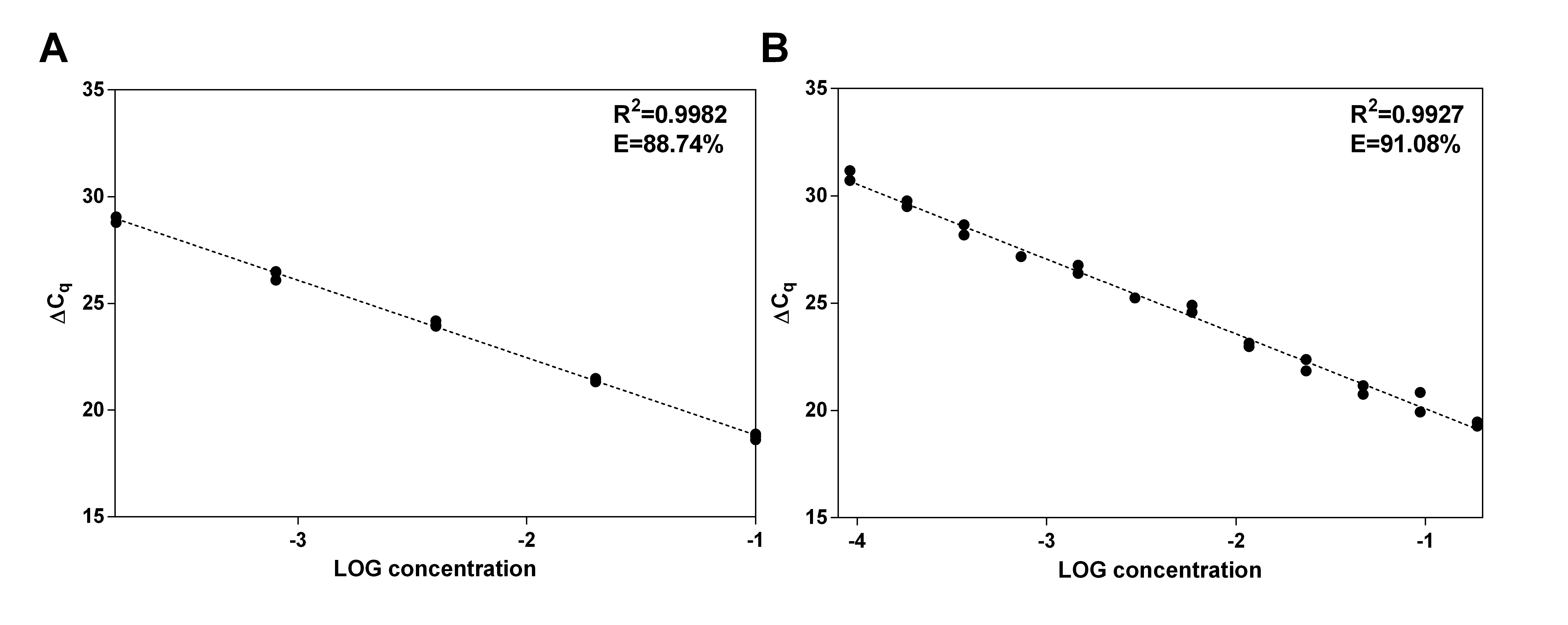


**Online Resource 3** qPCR standard curves based on serial dilution of DNA from of: A. *S. latissima* with the primer pair CG64 and CG65. B. *L. elsbetiae* with the primer pair LelsITS1-F2 and LelsITS1-R2


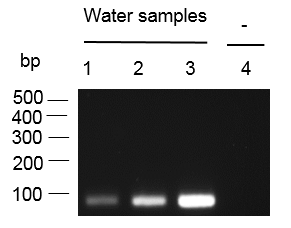


**Online Resource 4** Agarose gel (2.5%) of qPCR products from 3 sea water samples (1-3) and a negative control (autoclaved milliQ H_2_O, 4), amplified with the primer pair LelsITS1F2 and LelsITS1R2. Weight marker: SmartLadder SF (Eurogentec, Belgium)

**Online Resource 5** Statistic table: Distribution of endophyte DNA in field sporophytes (Kruskal-Wallis Test)

| Kruskal-Wallis | Chi^2^ | df | p-value |
| --- | --- | --- | --- |
|  | 36,201 | 3 | 0.001 |

**Online Resource 6** Statistic table: Other experiments (One-Way ANOVA)

| One-Way ANOVA | F | df | p-value |
| --- | --- | --- | --- |
| Young kelps | 2.737 | 4 | 0.04 |
| Natural infection | 12.653 | 1 | 0.001 |
| Seasonal variation | 7.15 | 8 | 0.001 |
| Geographic variation | 4.877 | 2 | 0.01 |
| Host Specificity | 9.126 | 2 | 0.001 |
